# Supplementary material for: Single-Molecule Fluorescence Probes Interactions between Photoactive Protein—Silver Nanowire Conjugate and Monolayer Graphene
Source: Int J Mol Sci. 2024 Apr 30;25(9):4873. doi: 10.3390/ijms25094873 (PMC11084953; doi:10.3390/ijms25094873)
Supplement: Supplementary file 1 [file ijms-25-04873-s001.zip › ijms-2966372-supplementary.pdf]

Single-Molecule Fluorescence Probes Interactions between Photoactive Protein—Silver Nanowire Conjugate and Monolayer Graphene

Kamil Wiwatowski [1], Karolina Sulowska [1,2] and Sebastian Mackowski [1,*]

[1] Nanophotonics Group, Institute of Physics, Faculty of Physics, Astronomy and Informatics, Nicolaus Copernicus University, Grudziadzka 5, 87-100 Torun, Poland; kamwiw@gmail.com (K.W.); sulowska@fizyka.umk.pl (K.S.)

[2] Institute of Advanced Materials, Wroclaw University of Science and Technology, Wybrzeze Wyspianskiego 27, 50-370 Wroclaw, Poland

* Correspondence: mackowski@fizyka.umk.pl

Supplementary material

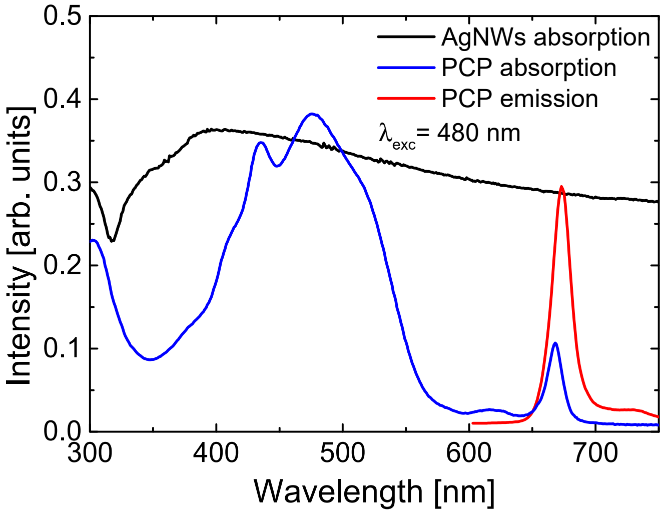

Figure S1. Absorption spectra of silver nanowires (black) and PCP in solution (blue), together with the fluorescence emission of the PCP solution upon excitation at 480 nm (red).

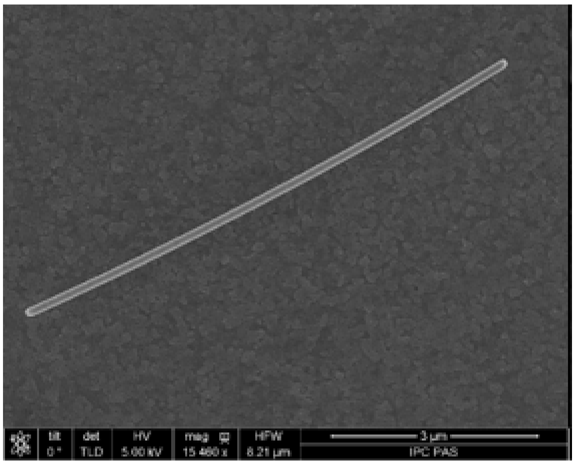

Figure S2. Scanning electron microscopy image of a single silver nanowire.

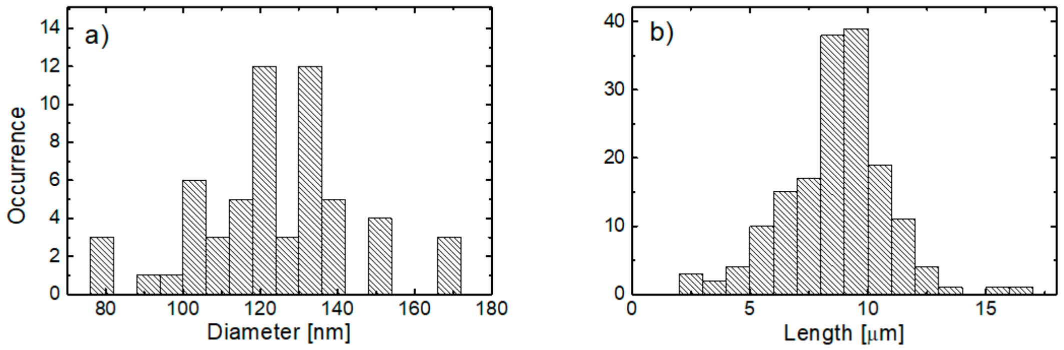

Figure S3. (a) Histogram of diameters of silver nanowires, and (b) histogram of lengths of silver nanowires.

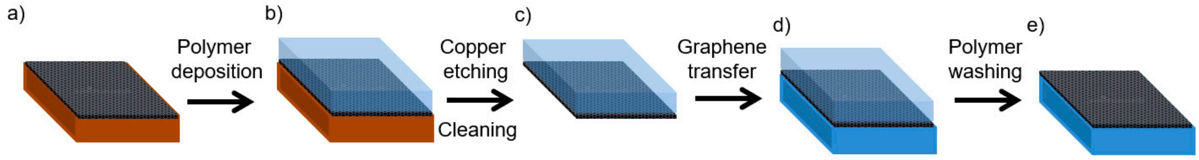

Figure S4. The scheme of developed graphene transfer method on arbitrary substrate, based on polymer assisted transfer (PAT) procedure. a) CVD graphene on copper foil, b) graphene covered with polymer, c) etching of copper and polymer-graphene stack cleaning, d) cleaned polymer-graphene stack transferred on selected substrate, e) washing of polymer.

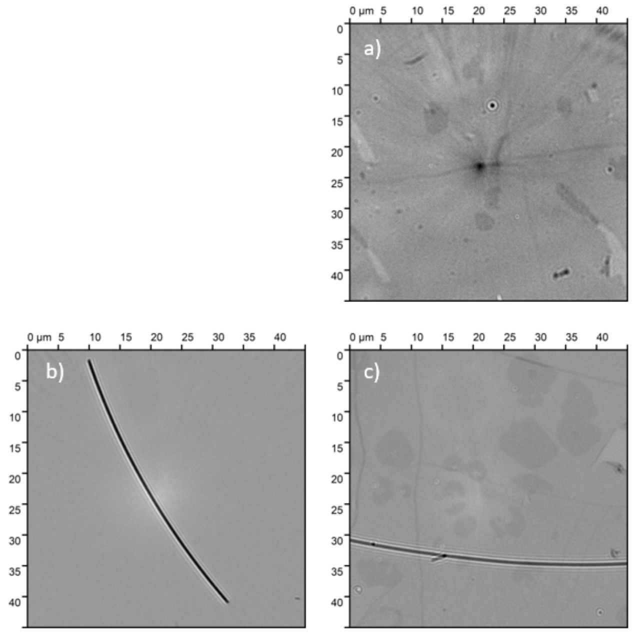

Figure S5. Transmission images of single PCP: a) on graphene, b) conjugated with AgNWs on glass and c) conjugated with AgNWs on graphene.

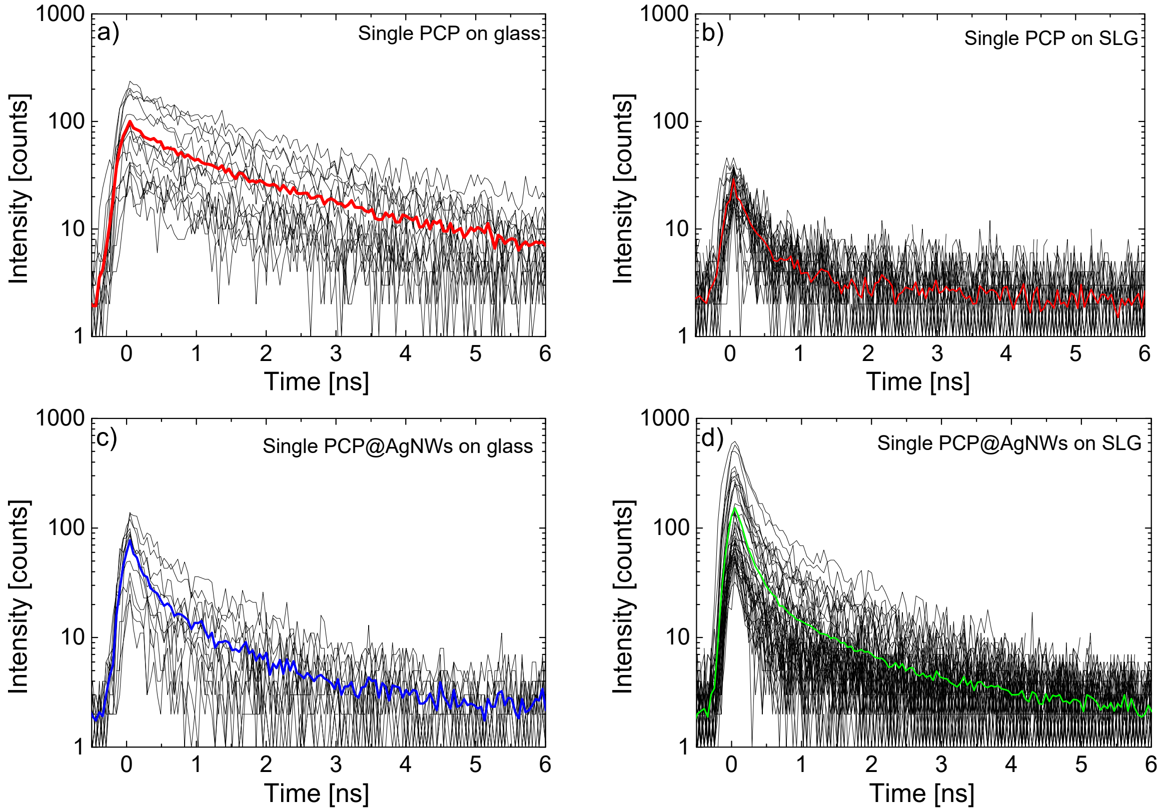

Figure S6. All time-resolved data measured with home-built confocal fluorescence microscope, together with corresponding averaged curves. The data is presented as follows: a) single PCP on glass, b) single PCP on graphene, c) single PCP@AgNWs on glass and d) single PCP@AgNWs on graphene.

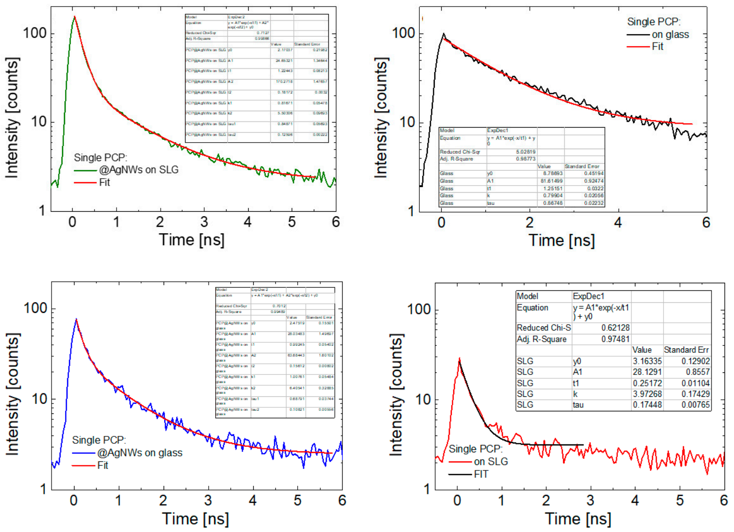

Figure S7. All time-resolved data measured with home-built confocal fluorescence microscope, together with corresponding averaged curves. The data is presented as follows: a) single PCP on glass, b) single PCP on graphene, c) single PCP@AgNWs on glass and d) single PCP@AgNWs on graphene.
